# Healthcare services gap analysis: a supply capture and demand forecast modelling, Dubai 2018–2030

**DOI:** 10.1186/s12913-023-09401-y

**Published:** 2023-05-10

**Authors:** Nahed Monsef, Eldaw Suliman, Elham Ashkar, Hamid Yahay Hussain

**Affiliations:** 1 Strategy & Governance Department, Strategy & Corporate Development Sector, Dubai Health Authority, Dubai, United Arab Emirates; 2 Research & Data Analyses Department, Strategy & Corporate Development Sector, Dubai Health Authority, Dubai, United Arab Emirates

**Keywords:** Capacity plan, Supply–demand analysis, Dubai, 2018–2030

## Abstract

**Background:**

Health systems aim to provide a range of services to meet the growing demand of Dubai's heathcare system aims to provide a range of services to meet the growing demand of its population health needs and to ensure that standards of easy access, quality, equity and responsiveness are maintained. Dubai Health Authority (DHA) uses health services planning tools to assess the health needs of its population and sets priorities and effective regulatory strategies to achieve equilibrium of supply and demand of healthcare services and ensure adequate healthcare services are available, in terms of both quality and quantity. This study aims to measure the gap between demand and supply in health care services in Dubai at the baseline and to forecast the gap size and type (according to medical specialty, key medical planning units and geographical area) till 2030. The specific consequential aim includes identification of appropriate strategic directions for regulation, licensing, policies, insurance.

**Methodology:**

The supply of healthcare services, professionals and medical equipment is captured through a census of all healthcare facilities licensed for practice in the Emirate of Dubai. The demand is estimated using a need based approach, where demand for episodes of medical care are estimated by age and gender and aligned to the internationally defined diagnosis related groups (IR-DGRs). The estimated episodes are then forecasted into the future, until 2030, using three scenarios of population growth (high, medium and low) for the emirate of Dubai. The captured supply and forecasted demand has been categorized into eight key health-planning units (KPUs) to allow for understanding of the population healthcare service needs by main service categories. Using a software for health services planning, a gap analysis between supply and demand is conducted till year 2030.

**Results:**

The results revealed a current and expected undersupply and oversupply for some healthcare services by medical specialty and geographical area of the Emirate. By 2030, the largest gaps exists in acute beds, which would require 1,590 additional beds, for acute-same day beds, an additional 1575 beds, for outpatient consultation rooms, an additional 2,160 consultation rooms, for emergency department, an additional 107 emergency bays, and for long-term care and rehabilitation beds, an additional 675 beds. The top specialty needs for these categories include cardiology, orthopedics, rheumatology, psychiatry, pediatric medicine & surgery, gastroenterology, hematology & oncology, renal medicine, primary care, respiratory medicine, endocrinology, rehabilitation and long-term care.

**Conclusions:**

There is an existing and growing requirement to support the healthcare services capacity needs for the top service lines and geographical areas with the largest gaps. Future licensing is required to ensure that new facilities are geographically distributed in a balanced way, and requests for licensing that create or augment oversupply should be avoided.

**Supplementary Information:**

The online version contains supplementary material available at 10.1186/s12913-023-09401-y.

## Introduction

Healthcare service planning is a critically important strategic practice in healthcare to ensure the overall health needs of a population are met through effective planning and allocation of health resources. Essentially, understanding the needs of the population through a validated systematic planning process to ultimately determine the best means of addressing the population health service gaps and future health requirements [1]. Due to the complexity and nature of healthcare systems and provision of health services, particularly in a rapidly growing city like Dubai, it is important to examine many contributing factors that impact health service planning to ensure the delivery of quality care to patients [2].

High returns planning should refer to the opportunities and constraints that arise from the market segments. Thus, organizational segmentation in health care is linked to the urgency, severity as well as disease types, delivery models, and population subgroups. Without proper health, services planning potential conflicts on priorities, goals, and performance metrics may be encountered. That is why managerial visions are importantly required to decide on activities for integration of healthcare services. Moreover, the pressure of high population growth of Dubai (growing at 4.3% annually) which is fueled by large influx of expatriate workforce in all sectors of the economy has resulted in several new residential areas with no healthcare services nearby. The most populated areas with rapid residential expansion and lack of close proximity healthcare services are in Deira, Nahda, Jumeirah, Dubai land and Jebel Ali areas [3].

Over the past 70+ years, Dubai shows an evolution of expanding healthcare services capacity, adoption of more advanced technology, and policy setting that have shaped the current healthcare system. Starting from a small healthcare center in Al Ras, followed by the establishment of five government owned hospitals, 12 Primary Healthcare Centers (PHC), 48 private hospitals and around 4000 health facilities. This is in addition to a significant move towards universal health insurance coverage in 2013. The main driver for this enormous expansion in healthcare capacity was due mainly to the fast-growing population in Dubai. The aim of this study is to better understand the most critical gaps in health services and identify the strategic direction to address these gaps in the upcoming years.

As we continue to see rapid changes in medical advancements, increased healthcare investments, a rise in unique patient needs, and utilization of health services it is critical that strategic healthcare service planning is conducted early to ensure our healthcare infrastructure is capable of meeting current and future patient needs [4]. However, even many high-performing health systems are faced with the challenges of ensuring the right balance or equilibrium of health services where supply should equal demand (future needs of the population). This will help eliminating any duplication of services and rationalization of appropriate services based on patient and geographical needs. A periodic study was conducted to understand the current healthcare sector demand, supply and future gaps in healthcare services [5–7].

Capacity planning exercises are a useful tool for evidence-based information required to assess and identify gaps in healthcare services. The consulting company that worked with DHA to conduct the capacity planning study is the Total Alliance Health Partners International (THAPI), which has conducted several capacity planning exercises across many healthcare systems including UAE, Singapore, Australia, the Philippines & Papua New Guinea. However, globally many health care systems and organizations conduct a similar exercise for multiple purposes including planning for the future healthcare workforce, allocation of medical services, and/or specific service line/specialty (i.e. emergency rooms, etc.) [8–10].

### Research aims & objectives

To help estimate and plan for the future needs of health care services based on global best practices, population growth, and geographical access to healthcare services, to ensure a balanced supply of healthcare services and workforce across the Emirate of Dubai.

### Objectives

The specific objective of this research paper is to measure the gap between demand and supply in health care services in the emirate of Dubai, identify priority gaps and set strategic goals to bridge these gaps through new service delivery frameworks, infrastructure developments, and workforce planning to accommodate Dubai’s healthcare services need till year 2030.

### Research context & framework

#### Context

The Dubai Clinical Services Capacity Plan 2018–2030 (DCSCP) [11] was developed and undertaken to provide evidence to support and deliver the strategic priorities of the Dubai Health Sector Strategy 2022–2026. The DCSCP will identify and validate the current inventory (supply) of healthcare services across nine geographical sectors of Dubai; project the demand for healthcare services till 2030; analyze the supply gap and identify short and long-term healthcare service priorities for Dubai.

### Healthcare services supply and demand analytical framework

**Fig. 1:**
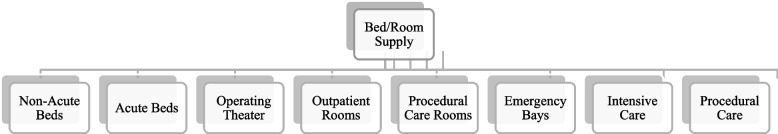
Health care services key planning units (KPUs)

The Dubai capacity planning current and planned supply enumeration and current and future demand estimation is conducted based on the premises of eight healthcare services key planning units (KPUs) as illustrated in Fig. 1 below.

**Fig. 2:**
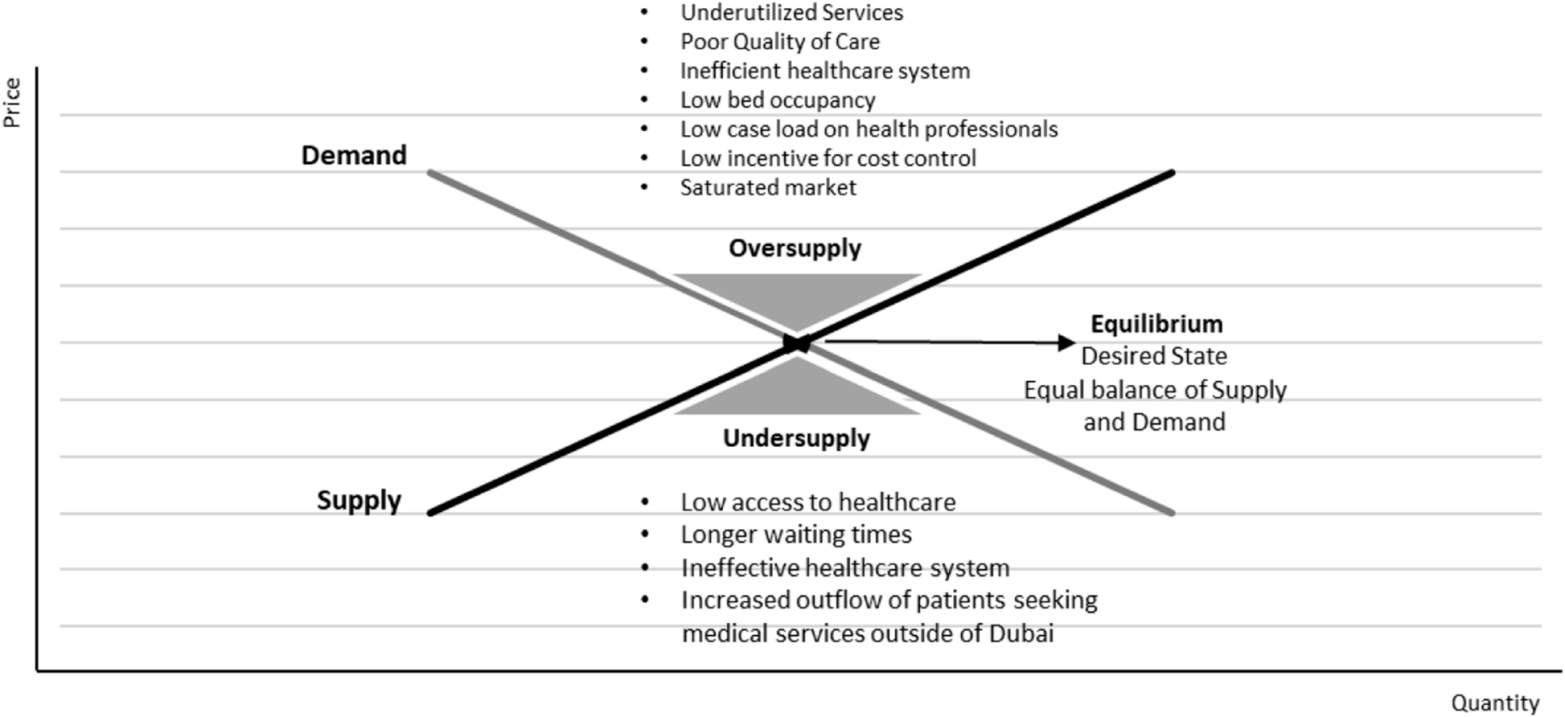
Dynamic of supply and demand and gaps analysis

The strategic desired state of the Dubai health sector is to achieve an equilibrium in supply and demand of healthcare services. Both oversupply and undersupply are undesirable situations in good healthcare system. As Fig. 2 shows, an oversupply may lead to poor quality of healthcare services resulting from underutilization and low caseload of services. In addition, oversupply may lead to an inefficient healthcare system where hospital beds are under occupied and healthcare service prices are distorted. On the other hand, undersupply, which is a reflection of a gap in services available, may lead to longer waiting times, restricted or delayed access to care and more demand for healthcare from abroad, which will augment the existing pressure on Dubai government health sector budget.

## Methodology

As mentioned above, supply of healthcare services in Dubai is captured through a structured healthcare facility survey, which included six distinct healthcare establishments, namely; (hospitals, clinics, centers, day surgery centers, rehabilitation centers, & diagnostic centers.). The demand for healthcare services is estimated as a need based demand with two main factors, age and gender, are considered the derivers of health seeking behavior and demand for healthcare. Disease related groups (DRGs) by age and gender are used to estimate the expected demand for healthcare services for UAE nationals and expats separately to account for the differences in access to care between these two groups. Then, demand is segregated by region according to the nine regions of the emirate of Dubai.

The DRGs used are from an international standard DRGs driven from healthcare system with reliable, accurate and complete coding of diseases (namely, Australia and USA). After the expected demand for healthcare services is estimated, then an adjustment for the burden of disease differences between the two reference countries as well as the socioeconomic differences was conducted to arrive at a more refined demand for healthcare for the emirate of Dubai. The demand thus was estimated through four main steps: (Need, Demand, Activity and Outcome). The mathematical equations used to estimate the demand are stated below:

### Same day place

As defined by the DCSCP [11] the number of beds or chairs required for a projected volume of same-day episodes is calculated as per the formula below:

$$\frac{\sum \mathrm{stay\, period\, days}/248}{1.5}$$

where, the stay period days is the sum of the patient same day stay periods over 248 working days each year (allowing 12 weekday holidays), divided by an expected 1.5 patient use for each KPU in the same day.

### Overnight bed

As defined by the DCSCP [11]), the number of beds required for a expected volume of overnight episodes is given as per the formula below;

$$\frac{\sum \mathrm{stay\, period\, days}/365}{0.7}$$

where, the stay period days is the sum of overnight patient stay period days for a year, over 365 days per year and 70% occupancy rate as per the Dubai’s acceptable occupancy provision.

### Operating theatre

As defined by the DCSCP [11], the number of operating theatres required for an expected volume of operations is calculated as per the formula below;

$$\frac{\sum \mathrm{stay\, period\, minutes}/60/24/365}{Occupancy}$$

where, the stay period minutes are the sum of operational stay minutes, over 60 min per hour and 24 h in day over 365 days in the year adjusted 70% the occupancy rate for elective and 45% occupancy for emergency. This allows for greater demand variation for emergency surgeries.

### Outpatient consultation room

As defined by the DCSCP [11], the number of consultation rooms required for an expected volume of outpatient visits is calculated as per the formula below;

$$\frac{\sum \mathrm{visits}\times \mathrm{standard\, consultation\, time}/60/8/248}{0.7}$$

where, the standard consultation time is measured in minutes for each visit, over 60 min per hour, over 8 h in a typical working day and 248 working days in a year (excluding holidays and weekends), assuming 70% the room occupancy rate.

### Procedural unit

As defined by the DCSCP [11], the number of equipment required for an expected volume of procedures is calculated as per the formula below;

$$\frac{\sum \mathrm{occurences}\times \mathrm{standard\, procedure\, time}/60/8/248}{0.7}$$

where, the standard procedure time is the number of minutes for a single procedure over 60 min per hour, over 8 h a day and 248 days in a year (excluding holidays and weekends), assuming 70% room/place occupancy rate.

### Emergency department bed

As defined by the DCSCP [11] the number of Emergency Department (ED) cubicles required for an expected volume of emergency visits is calculated as per the formula below;

$$\frac{\sum \mathrm{stay\, period\, minutes}/60/24/365}{0.7}$$

where, the stay period minutes is the sum of emergency stay minutes, over 60 min per hour, 24 h a day over 365 days per year and assuming an occupancy rate of 70%.

### Intensive care bed

As defined by the DCSCP [11], the number of beds required for an expected volume of intensive care episodes is calculated as per the formula below;

$$\frac{\sum \mathrm{stay\, period\, days}/365}{0.7}$$

where, the stay period days are the sum of the intensive period days over 365 days in a year and assuming a 70% the occupancy rate.

### Full time equivalent (FTE)

As defined by the DCSCP [11], the number of FTE in health care professionals required for a specific study population is calculated as per the formula below;

$$\mathrm{FTE}\_\mathrm{r }\times \mathrm{ popcnt}\_\mathrm{s}$$

where, FTE_r is demand reference rate for FTE and popcnt_s is the study population.

Overnight Bed Estimate - The number of beds required for an expected volume of overnight episodes is calculated as per the formula below;

$$\frac{\sum \mathrm{ stay\, period\, days}/365}{0.7}$$

The equations below describes the methodology used to calculate the demand for healthcare services based on the eight KPUs

$$\frac{\sum \mathrm{ visits }\times \mathrm{ standard\, consultation\, time}/60/8/248}{0.7}$$

#### Data and statistics

The study has been carried out across all government and private healthcare sector facilities in Dubai, during 2017–2018. This is a full enumeration (census) with primary data collection from hospitals and healthcare centers across the emirate of Dubai. Data was collected using a carefully designed survey questionnaire developed by the Dubai Health Authority team in collaboration with the Total Alliance Health Partners International (TAHPI), a consulting firm working in the field of capacity planning across the globe. The data gathered through the survey was compiled and aggregated by KPUs and specialties and segmented by the Dubai’s nine geographical sectors. The collected data was validated using two main external data sources to assess the validity and completeness of the data. The two main sources included the health insurance reimbursement E-claims database and the DHA annual statistical book 2017. In addition, retrospective records review was also used for validation based on secondary data retrieved from electronic medical records of government facilities (Salama system), the emirate wide licensing system (Shyrian) as well as the electronic medical which contains partial data for public and private healthcare facilities (Nabdh system). During the validation process, an exclusion criterion was adopted, (missing, inadequate data records, old data - more than 10 years) were excluded.

**Operational definitions of variables** e.g. **Supply** is the total amount of healthcare services that are available for use by the populations. Health service **demand** uses a health service-planning module developed by TAHPI to estimate current and future demand for healthcare services among Dubai’s population segmented by age, gender, geographic sector, and nationality status [12, 13].

Progressing from population modeling to supply capture, and in sighting the demands modeling, and gap assessment, in low, medium and high scenarios. The low Demand scenario assumes 4% of population growth, the medium demand scenario assumes 5.3%, and high demand scenario 7.4%). Demand Modeling is used to estimate demand for the years 2018–2030. The gaps identified for each KPU are a result of the difference between supply and demand. Further adjustments were made, in consultation with subject matter experts from DHA, to ensure that results aligns with the relative utilization factors for Dubai after adjusting the international reference rates.

## Results

This section presents the results of the supply, demand and gaps at the KPU and specialty level for the 2018, 2020, 2025 and 2030. Graphical and tabular presentations of the gaps (oversupply /undersupply) are provided for each KPU.

The results show the priorities that will require new investment and development of the existing healthcare facilities and workforce to accommodate Dubai’s future health needs till 2030. The numbers presented in the results are vital information for conducting feasibility studies to support decision-making process for engaging in new investments.

**Fig. 3:**
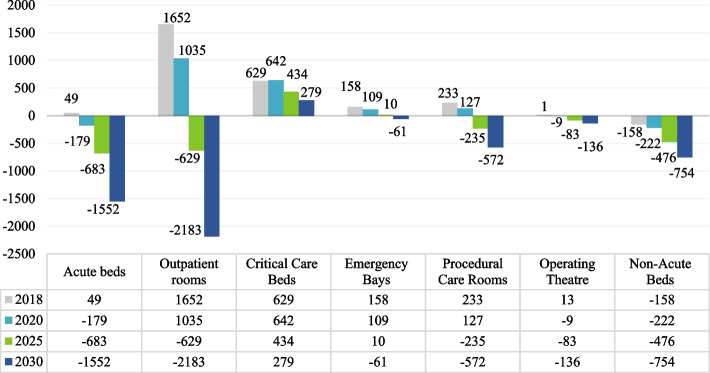
Gaps by KPUs. Note: a –ve sign shows the size of additional services required for a specified year and KPU

Figure 3 references the gaps by each key planning unit (KPU). Gap in acute beds is (−179) in 2020, (−683) in 2025 and (−1552) in 2030. The gap will be (−629) outpatient room in 2025, and (−2183) in 2030. Critical care beds reflected no gaps and witnessed a decreasing pattern in the bed number of 629 beds in 2018 reaching 279 beds in 2030. As for the Emergency Bays, the data revealed that it is +158 in 2018 and −61 in 2030. Procedural care showed +233 rooms in 2018 and -572 in 2030. The operating theater revealed that i + 13theaters in 2018 and −136 in 2030. For Non-acute beds, it shows −158 in 2018 and −754 in 2030.

**Fig. 4:**
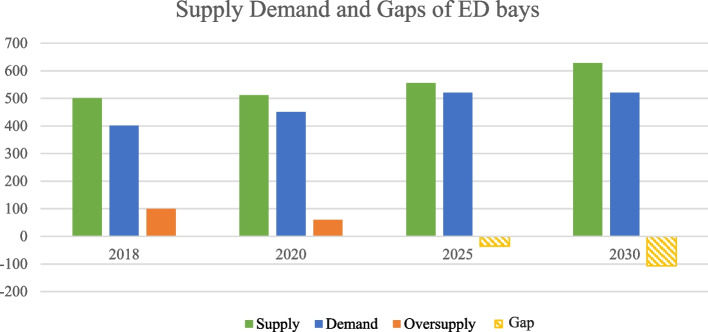
Supply, demand, and gaps of ED bays, 2018 to 2030

Figure 4 revealed that in 2018 there is an oversupply gap in ED Bays of about 100 (supply: 501, demand: 401), while in 2020, the oversupply gap reduced to 60 (supply 511, demand 541), as in 2025 there will be a demand gap of −35 (supply 556, demand 521). In addition, for 2030 there will be a larger demand gap of −107 bays (supply 628, demand 521).

**Fig. 5:**
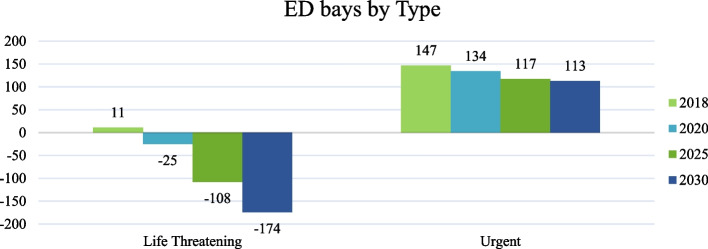
Gaps of ED bays by Types

Figure 5 showed that Emergency Department bays for life-threatening cases will show gaps starting from 2020 at −25, in 2025 will be −108 and in 2030 will be −174 > As for the urgent service, no gaps will be available, as in 2018, it is estimated as 147, in 2020 it is 134, in 2025 it will be 117 and in 2030 it will be 113.

**Fig. 6:**
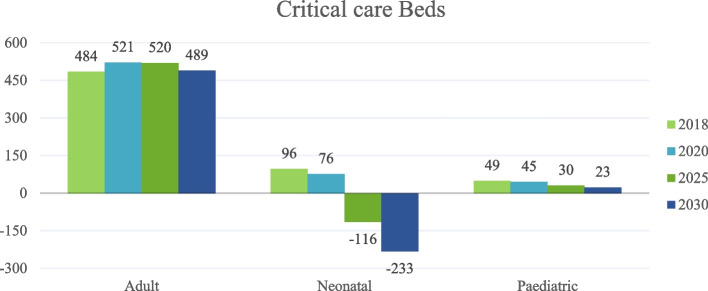
Gaps in critical care Beds

Figure 6 reflected that critical beds for adults will keep going on without gaps, 2018 was 404, 2020 was521, 2025 will be 520, and 2030 will be 489. As for the neonatal, there will be a gap of −116 in 2925 and −233 in 2030. As for pediatrics, no gaps were revealed in the trend 2018 was 49, 2020 was 45, in 2025 it was 30 and in 2030, it will be 23.

**Fig. 7:**
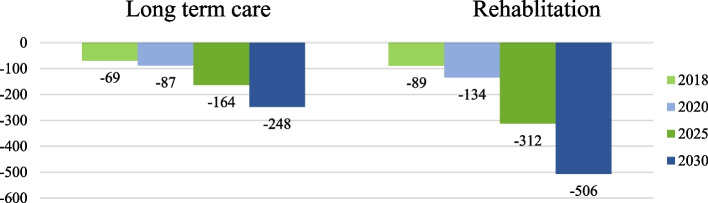
Supply-demand gaps in Long-term care and rehabilitation

Figure 7 showed that Supply demands Gaps in rehabilitation and long-term care. Concern with the Long term, care revealed gaps of −69 in 2018, −87 in 2020, −164 in 2025, and −284 in 2030. As for Rehabilitation, the gaps are −89 in 2018, −134 in 2020, −312 in 2025, and −505 in 2030.

## Discussion

This study identifies the gaps between the demand for the healthcare services and the current and planned supply within the Emirate. The gap analysis was conducted by calculating by the difference between demand and supply, where a deficit indicates a need for additional capacity and a surplus suggests an opportunity for reallocation of capacity to geographical areas with shortage in services [11]. The analysis identifies priorities for the development of healthcare services needed and workforce required until 2030. A study in Brazil [14] concluded that there is a true necessity of identifying priority areas for the future of the national healthcare system, addressing the population’s health needs and problems, and providing options for government agencies and investors to act. The current study can be used to provide support for the development and monitoring of effective health policies for national, regional, and local intervention as the case shown in other studies [15], which revealed that mature ecosystem service, shall effectively contribute to adequate services provision, and unique facilitates landscape planning by structuring problems and providing data for decisions making. All healthcare service gaps are defined by key planning units (e.g. beds, chairs, operating theatres, consultations rooms, full-time equivalents) using local context assumptions such as occupancy, turnover, and operational days. Key planning units used in this study describe infrastructure and workforce capacity required for a healthcare delivery system in a similar way to the WHO approach [16]. For external validation of the results, Singapore is chosen for comparison because it is a high-income country with a young population of expats like Dubai. In addition, they also have the same healthcare policies and current healthcare structure as Dubai [17]. Comparing our results to a study conducted in Singapore, we found a surplus in emergency room beds. This may be due to the large capacity that was built at the time of COVID. The demand for acute healthcare services remains high and the capacity remains tight and this finding in fact reflecting the two countries contexts similarities in terms of health care policies, health care structure, economic growth and other elements of health care delivery models [18–20].

The current study revealed that the critical care beds for adults in Dubai would experience significant oversupply capacity for the years 2018–2030, comparing to lower oversupply capacity for pediatrics group. There will be only undersupply gaps encountered in the critical care beds for neonates for the years 2025 and 2030, in comparing to another study [18] which revealed that the critical care capacity in many southeast Asia countries vary widely and it is significantly lower in low- and lower-middle-income than in upper-middle-income and high-income countries regional wise.

The current study revealed that an undersupply gap currently exists in long-term care and will continue into 2025 and 2030, resulting in short- and long-term challenges of matching resource capacity with uncertain demand for hospitals and other healthcare providers, if not addressed [18, 21, 22]. While rehabilitation capacity in Dubai is currently experiencing an undersupply gap starting in 2018, the gap is expected to be continuously increase in 2022, 2025, and 2030. These findings are similar to Saudi Arabia’s study findings [23, 24] that stated that the clinical practices regarding patient’s rehabilitation and health restoration involve various challenges and highly complex clinical interventions.

## Conclusion

Dubai’s population is experiencing a rapid growth at 3.9 percentage points, which is approximately more than four folds the world’s current average growth of 1.1% per annum as per Dubai Statistics Report [25]. This growth has caused a progressive shift in its age, gender, and nationality composition pattern. In addition, the epidemiological profile has impacted the utilization pattern of health care services. Based on these changes the health care needs are increasing at faster pace, hence requiring more efficient utilization of the existing healthcare services infrastructure and further planning and investment promotion to attract investors to invest in the projected specialty gaps [11]. For example, expanding long-term care capacity and improving its model of care will reduce intensive care bed utilization by patients requiring long-term assistance, which is one of Dubai’s largest contributors to the over utilization of acute beds by long-term patients. There is also a growing demand and gap in neonatal ICU beds, which can be managed by promoting targeted investment through certificate of needs and incentive schemes. This growth in demand is largely due to increased neonatal survival rates resulting from technological developments and increased number of high-risk child births [26]. There is a need to review licensing policies and requirements to specify the suitable minimum critical care beds at the time of licensing stage to ensure that beds are approved and licensed to address the expected demand and the international bed planning benchmarks.

It is also recommended that at the micro level each hospital in the Emirate of Dubai conduct resource optimization exercise for better planning of hospital immediate and future needs. Similar practices for hospital resource optimization exist globally such as the National Health Services (NHS) hybrid forecasting-simulation–optimization model [27].

There is a growing need for non-acute and long-term care capacity, which requires strategic licensing and funding incentives to attract investment in rehabilitation, transitional and long-term care services. Public and private partnership opportunities should be encouraged to support funding, design, build and operate long-term care, rehabilitation, post-acute care services to address the insufficient supply and meet the growing demand. These models of care should be integrated across the continuum of care. This includes the addressing the needs of the patients requiring ongoing continuous management and support with daily living activities.

Additional emergency bays for life-threatening cases are in an immediate need till 2030, as manifested by the high levels of emergency department activity at public hospitals where most life-threatening care is provided in Dubai. To reduce the burden on the public facilities, it is recommended that life-threatening capacity be increased in private facilities where patients are presenting in large volumes (80% of the healthcare services are provided by the private sector facilities). Any additional capacity, whether provided by the public or private sector, should be reallocated to rapidly respond to and effectively identify and treat patients who are at a high probability of dying to avert premature deaths.

Clearly defined insurance coverage and reimbursement framework for rehabilitation and long-term care are required to improve service availability and accessibility in response to the increasing demand, particularly for expats. Exploring options for community-based and outreach non-acute care to mitigate facility capacity gaps is warranted. A large component of rehabilitation and long-term care can be cost-effective if delivered in community and home care settings. Community and home settings can leverage technology and advanced wearable systems to manage and remotely monitor patients through the aid of telemedicine and virtual solutions. Linking reimbursement to treatment outcomes is currently underway, via the implementation of DRGs, that will increase the demand for step down and long term care facilities due to pay for performance/outcomes and length of stay caps on acute care.

### Policy implications

Due to the growing demand for healthcare services in the region, (MENA and GCC Region) it is imperative for the future health care planning strategies to shift towards adopting innovative and smart technologies to improve the care delivered and focus on prevention to maintain an optimal balance of supply and demand of healthcare services in Dubai. Licensing policies should limit oversupply, which could result in redundancy and diminish the quality of healthcare services.

### Study strengths & limitations

**Study strength** is reflected through novel data, which has been carefully collected along with strong statistical analysis and deep sighing of the results. In addition, the supply capture used a full enumeration approach (Census) for all health care facilities in the Emirate of Dubai, i.e. data on supply of healthcare services was collected from each facility licensed and operating in Dubai.

#### Limitations

Adjusting to high standard health system indicators is somehow complex procedure that may hide some relative potential unintentional errors, forecasting analysis may give relative but not robust precision. However, to overcome this limitation, the study used a sensitivity analysis to validate the results.

## Supplementary Information

**Additional file 1.**

## Data Availability

All data generated or analysed during this study are included in this published article Dubai Clinical Services Capacity Plan 2018–2030 and can be accessed via https://www.dha.gov.ae/uploads/122021/e9b6b25d-1339-4f2e-8fbf-2b8ea3217315.pdf. The raw datasets generated and/or analysed during the current study are not publicly available due to the Dubai Health Authority raw data sharing policy and rules. However, data are available from the corresponding author upon responsible request with permission from the Dubai Health Authority given that the requestor signs a non-disclosure agreement (NDA).

## Abbreviations

DHA: Dubai Health Authority (DHA)
DRG: Disease Related Groups
DCSCP: Dubai Clinical Services Capacity Plan 2018–2030
ED: Emergency Department
KPU: Key Planning Unit
THAPI: Total Alliance Health Partners International
UAE: United Arab Emirates
WHO: World Health Organization

## References

[1] Reddy KS, Patel V, Jha P, Paul VK, Kumar AS, Dandona L (2011). Towards achievement of universal health care in India by 2020: a call to action. Lancet.

[2] Cilliers P (2001). Boundaries, hierarchies, and networks in complex systems. Int J Innov Manag.

[3] Taha A, Rodríguez-Vega G. Planning and Budgeting. Critical Care Administration. SpringerCitations - Details Page (springernature.com). 2020.

[4] Eternity HH (2020). Understanding the impacts of NPM and proposed solutions to the healthcare system reforms in Indonesia: the case of BPJS Get access Arrow. Health Policy Plan.

[5] Dandonaab L, Katochc VM, Dandonaa R (2011). Research to achieve health care for all in India. Lancet J.

[6] Sheikh JI, Cheema S, Chaabna K, Lowenfels AB, Mamtani R (2019). Capacity building in health care professions within the Gulf cooperation council countries: paving the way forward. BMC Med Educ.

[7] Draft global strategy on human resources for health: workforce 2030. http://apps.who.int/gb/ebwha/pdf_files/WHA69/A69_38-en.pdf. Accessed 12 Mar 2019.

[8] Global Health Observatory data repository. http://apps.who.int/gho/data/view.main.92100. Accessed 12 Mar 2019.

[9] Chana K, Cheema S, Mamtani R (2017). Migrants, healthy worker effect, and mortality trends in the gulf cooperation council countries. PLoS One.

[10] Fikri M (2017). Roadmap 2017–2021: stronger organization and better response to the needs of member states in the eastern Mediterranean region. East Mediator Health J.

[11] Dubai Clinical Services Capacity Plan 2018 2030. https://www.dha.gov.ae/uploads/122021/e9b6b25d-1339-4f2e-8fbf-2b8ea3217315.pdf. Cited 06/12/2022.

[12] Charlesworth A, Lafond S (2017). Shifting from undersupply to oversupply: does NHS workforce planning need a paradigm shift?. Econ Aff.

[13] Heenan LDB (1980). Health service planning and projected population change: some observations for New Zealand. Soc Sci Med D Med Geogr.

[14] Requia WJ, Kondo EK, Adams MD, Gold DR, Struchiner CJ (2020). Risk of the Brazilian health care system over 5572 municipalities to exceed health care capacity due to the 2019 novel coronavirus (COVID-19). Sci Total Environ.

[15] Fontana V, Radtke A, Fedrigotti VB, Tappeiner U, Tasser E, Zerbe S, Buchholz T (2013). Comparing land-use alternatives: using the ecosystem services concept to define a multi-criteria decision analysis. Ecol Econ.

[16] WHO Handbook 2010. Monitoring the building blocks of health systems: a handbook of indicators and their measurement strategies. ISBN 978 92 4 156405 2. https://apps.who.int/iris/bitstream/handle/10665/258734/9789241564052-eng.pdf. Accessed 4 Apr 2022.

[17] Bed capacity plan report. Ministry of Health Singapore; 2014. Visted 30/03/2023 1 pm. https://www.moh.gov.sg/news-highlights/details/bed-capacity-and-emergency-departments. Accessed 4 Apr 2022.

[18] Phua J, Faruq MO, Kulkarni AP, Redjeki IS, Detleuxay K, Mendsaikhan N, Sann KK, Shrestha BR, Hashmi M, Palo JE, Haniffa R, Wang C, Hashemian SMR, Konkayev A, Mat Nor MB, Patjanasoontorn BS, Nafees KMK, Ling L, Nishimura M, Al Bahrani MJ, Arabi YM, Lim C-M, Fang W-F (2020). Critical care bed capacity in Asian countries and regions. Crit Care Med.

[19] Ansah JP, Ahmad S, Lee LH, Shen Y, Ong MEH, Matchar DB (2021). Modeling emergency department crowding: restoring the balance between demand for and supply of emergency medicine. PLoS One.

[20] He L, Madathil SC, Oberoi A, Servis G, Khasawneh MT (2019). A systematic review of research design and modeling techniques in inpatient bed management. Comput Ind Eng.

[21] Palmer GI, Harper P, Knight V, Brooks C (2021). Modelling changes in healthcare demand through geographic data extrapolation. Health Syst.

[22] Geerts J, Willemé P. Projections of use and supply of long-term care in Europe: policy implications. ENEPRI policy brief no. 12. https://deliverypdf.ssrn.com/delivery.php?. Visited on 09-04-2022.1 pm.

[23] Alsufyani AM (2020). Role of treatment theory and enablement theory for restoring health and rehabilitation in Saudi Arabia. Cureus.

[24] World Health Organization. International classification of functioning, disability, and health (ICF). 2018. https://www.who.int/classifications/icf/en/.

[25] Dubai Statistics center reports. https://www.dsc.gov.ae/en-us/Pages/default.aspx. Visited on 31^st^ March 2023, 12PM.

[26] Bener A, Abdulrazzaq YM, Dawodu A (1996). Sociodemographic risk factors associated with low birthweight in United Arab Emirates. J Biosoc Sci.

[27] Ordu M, Demir E, Davari S (2021). A hybrid analytical model for an entire hospital resource optimisation. Soft Comput.

