# Supplementary material for: Healthcare services gap analysis: a supply capture and demand forecast modelling, Dubai 2018–2030
Source: BMC Health Serv Res. 2023 May 10;23:468. doi: 10.1186/s12913-023-09401-y (PMC10173558; doi:10.1186/s12913-023-09401-y)
Supplement: Supplementary file 1 — Additional file 1. [file 12913_2023_9401_MOESM1_ESM.docx]

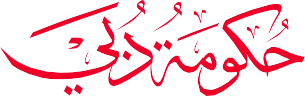


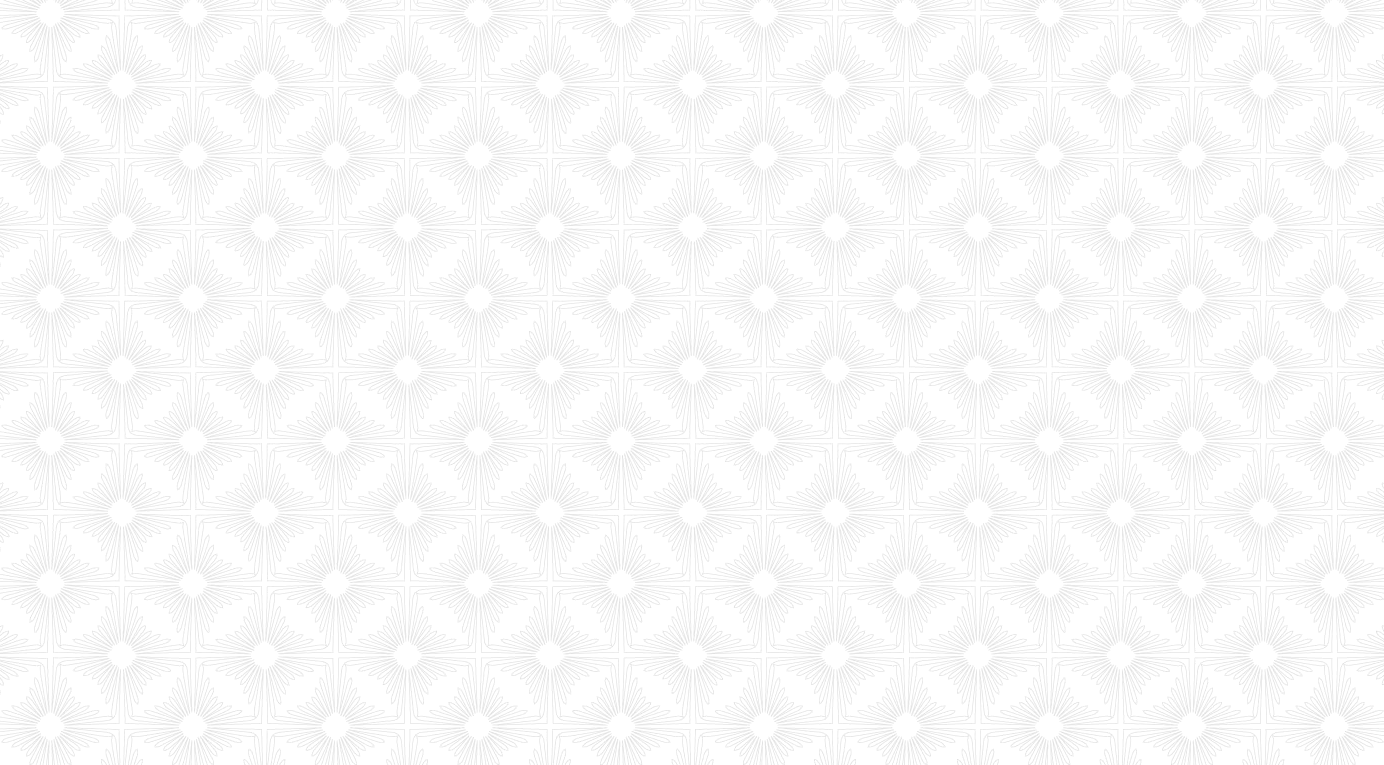

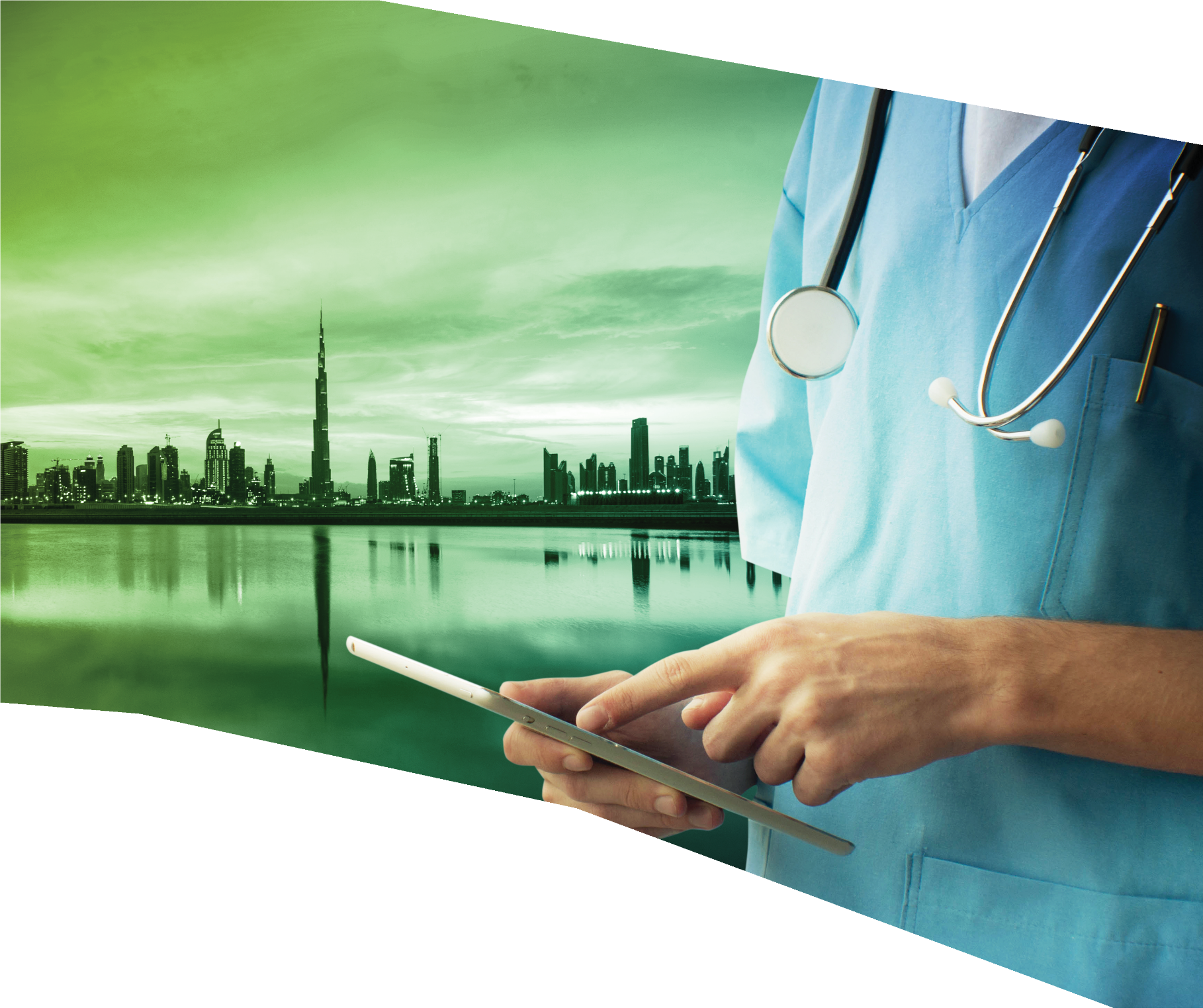


##
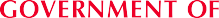

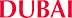
Supplementary File


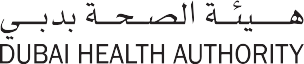


Figure 2: Dubai land sectors and sector size (km**2**)


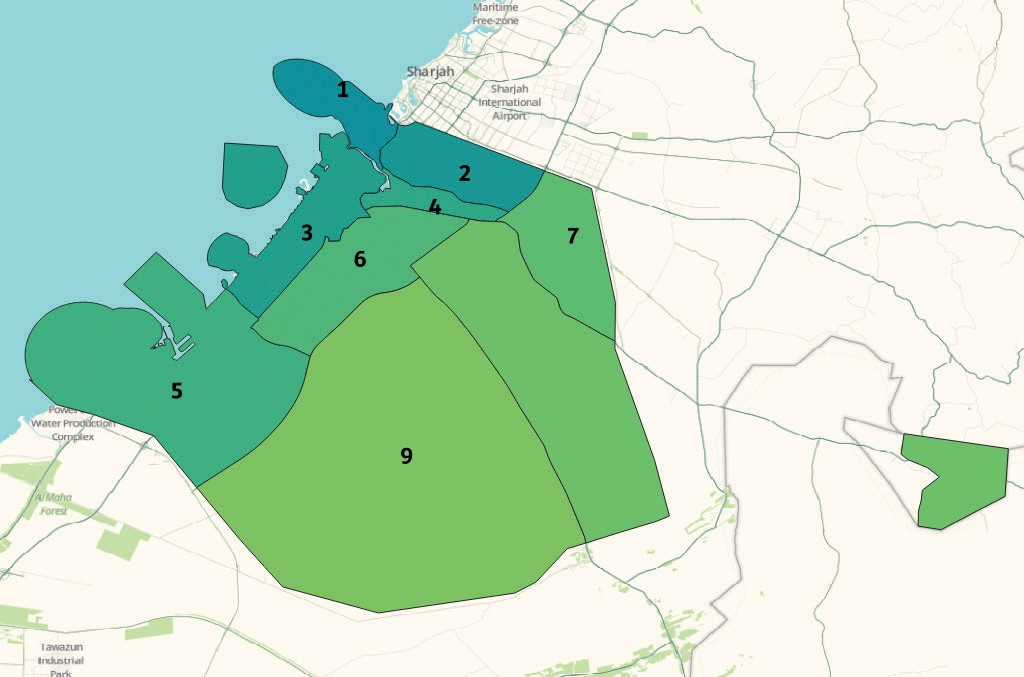

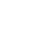

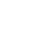

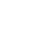

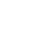

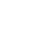

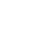

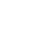

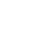

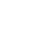


**1**

201

**2**

177

**3**

240

**4**

**6**

295

62

**7**

231

**5**

763

**8**

859

**9**

1678

Figure 1: Population projection scenarios, 2018 to 2030

6.0

Millions

#### 5.5


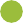

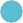

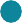

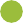

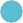

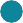

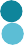

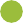

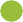


5.5

5.0

4.5

**Population**

4.0

3.5

3.0

2.5

#### 3.5 3.4

**3.1 3.3**

**4.6**

#### 4.1

**3.7**

**4.6**

#### 4.0

2018 2020 2022 2024 2026 2028 2030

High Medium Low


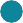

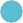

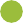


The **medium scenario** is used as the agreed projections for capacity planning, where the population is expected to increase on average 4% per annum from 3.1 million in 2018 to 4.6 million by 2030. For further information on population modelling, please refer to Chapter 8 – Appendix.

The accurate projection of health service demand is significantly influenced by a population’s age and gender structure with people in the high dependency age groups of 0-4 and 60+, and fertile women having higher service demand than other population components. Also, it is crucial that a clear understanding of the sector residency location and their expected growth is understood so that services and facilities can be estimate for locations of growth and need.

##### Sectors

Dubai is divided into nine geographic sectors to assist with the planning of the distribution of infrastructure capacity including healthcare facilities.

Population projections were conducted separately for each of the nine sectors using best fit probabilistic modelling from historical trends with 2006 as the base year (using sectorial population data reported from Dubai population bulletins and the 2012 Dubai Clinical Services Capacity Plan).

Table 3: Projected Population by Sector


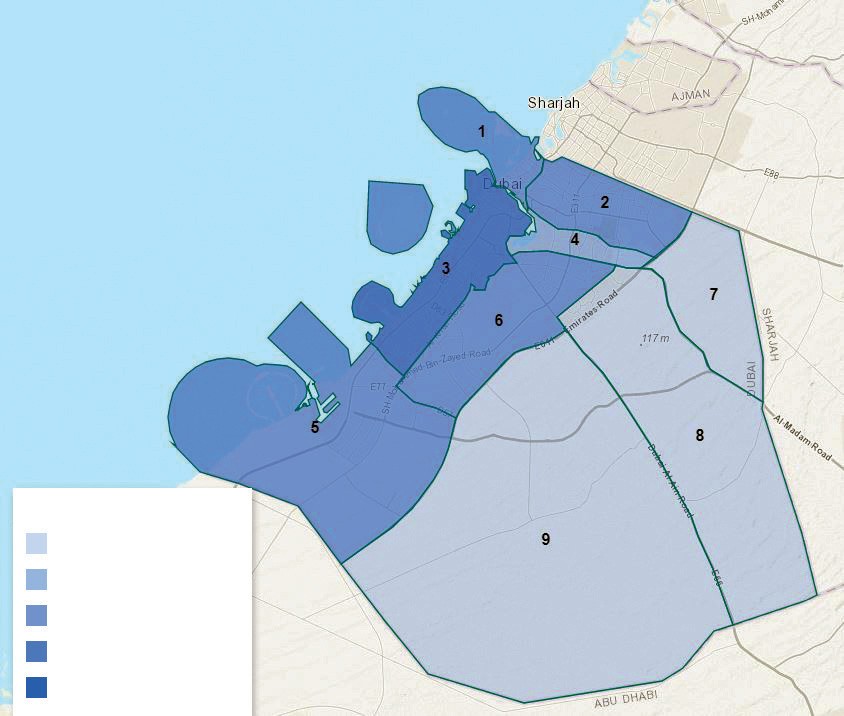

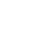

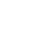

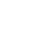

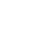

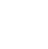

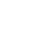


| **Sector** | **2018** | **2030** | **1**  **2**  **3 4 7**  **6**  **5 8**  **POPULATION**  <=30,000 **9**  30,000 to 100,000  100,000 to 500,000  500,000 to 1,000,000  > 1,000,000 |
| --- | --- | --- | --- |
| **1** | 588,138 | 911,122 |  |
| **2** | 627,734 | 760,842 |  |
| **3** | 1,172,732 | 1,814,567 |  |
| **4** | 54,917 | 84,610 |  |
| **5** | 417,222 | 647,127 |  |
| **6** | 177,935 | 275,983 |  |
| **7** | 11,499 | 17,786 |  |
| **8** | 40,080 | 62,027 |  |
| **9** | 9,743 | 15,066 |  |

Figure 3: Age group distribution, by sector, 2018

**Population** Thousands

Sector 1

Sector 2

Sector 3

Sector 4

Sector 5

Sector 6

Sector 7

Sector 8

Sector 9

0 200 400 600 800 1,000 1,200

0-19 20-39 40-59 60-79 80+

##### Age, gender and nationality profile

The population structure of Dubai displays a relatively young population and comprised by a majority of 67% males and 90% non-nationals.

Table 4: Medium scenario population profile, 2018 and 2030

|  | **Population** | **Nationals** | **Non-Nationals** | **Male** | **Female** |
| --- | --- | --- | --- | --- | --- |
| **2018** | **3,100,000** | 298,356 | 2,801,644 | 2,091,765 | 1,008,235 |
| **2030** | **4,589,131** | 414,625 | 4,174,506 | 2,967,911 | 1,623,220 |

The demand for healthcare is a needs-based demand that is sensitive to the age, gender and nationality composition of the population. This population composition has been analysed through the quantification of demand on all service types by per capita rates for each 5-year age group, gender and nationality category. This results in highly sensitive analysis on the population profile by dividing the population into 72 sub-categories, ensuring that demand is specific to different growth scenarios.

Figure 4: Dubai population by age, gender and nationality, 2018

85+

Female

Male

80-84

75-79

70-74

65-69

60-64

55-59

50-54

**Age group**

45-49

40-44

35-39

30-34

25-29

20-24

15-19

10-14

5-9

0-4

Nationals Non-Nationals

300 200 100 100 200 300 400 500 600

**Population** Thousands

Figure 5: Dubai population by age, gender and nationality, 2030

85+

Female

Male

80-84

75-79

70-74

65-69

60-64

55-59

50-54

**Age group**

45-49

40-44

35-39

30-34

25-29

20-24

15-19

10-14

5-9

0-4

Nationals Non-Nationals

300 200 100 100 200 300 400 500 600

**Population** Thousands

Table 10: Summary of current license workforce by facility type and category, headcount

| **Facility Type** | **Category** | | | | | **Total** |
| --- | --- | --- | --- | --- | --- | --- |
|  | **Medical** | **Nurses &**  **Midwives** | **Allied**  **Health** | **Dentist** | **Comp. Alt.**  **Medicine** |  |
| **Hospital/DSC** | 4,828 | 10,317 | 3,762 | 322 | 1 | **19,230** |
| **Outpatient Care Facility** | 2,951 | 5,361 | 2,253 | 1,798 | 196 | **12,559** |
| **Pharmaceutics** |  |  | 3,559 |  |  | **3,559** |
| **Others** | 75 | 649 | 623 |  |  | **1,347** |
| **Diagnostic Centres** | 102 | 24 | 546 |  |  | **672** |
| **Total** | **7,956** | **16,351** | **10,743** | **2,120** | **197** | **37,367** |
| **DHA 2017 headcount subtotal** | **1,847** | **4,645** | **N/A** | **178** | **N/A** |  |

Only 3% of the entire licensed healthcare workforce are UAE Nationals, with the largest majority from India and Philippines which contribute nearly 70% of the workforce by headcount.

Table 11: Top 10 largest proportion of workforce, by nationality and category

| **Nationality** | **Category** | | | | | **Total** |
| --- | --- | --- | --- | --- | --- | --- |
|  | **Medical** | **Nurses &**  **Midwives** | **Allied**  **Health** | **Dentist** | **Comp. Alt.**  **Medicine** |  |
| **India** | 30% | 50% | 49% | 32% | 51% | **45%** |
| **Philippines** | 1% | 39% | 18% | 2% | 0% | **23%** |
| **Egypt** | 11% | 1% | 5% | 6% | 2% | **4%** |
| **Pakistan** | 9% | 1% | 5% | 2% | 0% | **4%** |
| **UAE** | 7% | 0% | 3% | 5% | 2% | **3%** |
| **Syria** | 6% | 0% | 3% | 10% | 0% | **3%** |
| **Jordan** | 2% | 2% | 3% | 5% | 1% | **2%** |
| **Iran** | 3% | 1% | 1% | 5% | 1% | **2%** |
| **United Kingdom** | 4% | 1% | 1% | 2% | 7% | **2%** |
| **Sudan** | 4% | 0% | 2% | 1% | 1% | **1%** |

Across all workforce categories, the Dubai healthcare workforce has an average age of 37 years and is made up of 62% of females. A breakdown of workforce categories is shown in the table below, highlighting the differences in each measure between each category.

Table 12: Workforce average age and proportion by gender and by category

| **Measure** | **Category** | | | | | **Total** |
| --- | --- | --- | --- | --- | --- | --- |
|  | **Medical** | **Nurses &**  **Midwives** | **Allied**  **Health** | **Dentist** | **Comp. Alt.**  **Medicine** |  |
| **Average age** | 45 | 34 | 35 | 40 | 43 | **37** |
| **Males** | 58% | 15% | 44% | 51% | 36% | **35%** |
| **Females** | 40% | 83% | 50% | 48% | 57% | **62%** |
| **Unidentified** | 2% | 2% | 6% | 2% | 7% | **3%** |

**6.1 Acute Inpatient care (Overnight)**

Of the 5,169 licensed beds in Dubai, 671 are non-functional and 703 are allocated beds to same day care, which results in a supply of 3,795 functional acute overnight beds. Based on licensing applications and Expert Panel discussion, planned supply is expected to add an additional 861 beds by 2020 and a total of 1,162 beds by 2025.

| **Bed status** | **Number** |
| --- | --- |
| **Licensed beds** | **5,169** |
| - Non-functional beds | 671 |
| **Functional beds** | **4,498** |
| - Same day beds | 703 |
| **Acute overnight functional beds** | **3,795** |

Gap analysis shows that by 2025 there will be a gap of **311** acute overnight beds, which is projected to increase to

**1,590** by 2030.

Figure 15: Acute overnight bed gap, 2018 to 2030

Supply Demand Gap Oversupply


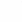


8,000

###### 6,547


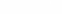

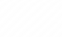


6,000

4,000

**Beds**

###### 3,795 4,656

**3,556**

**4,002**

**4,957**

**5,268**

**4,957**

2,000

0

-2,000

###### 239 654

**-311**

**-1,590**

2018 2020 2025 2030

The gap in 2025 can be addressed by utilising the non-functional beds currently built to address the requirements by specialty as shown below. Planned supply has been distributed by the proportion of gap for each specialty, assuming that future supply will likely address the service lines with the greatest requirements.

Table 13: Acute overnight bed gap by specialty, 2018 to 2030, sorted by 2030 gap

| **Specialty** | | **Supply** | | **Demand** | | | **Gap** | | |
| --- | --- | --- | --- | --- | --- | --- | --- | --- | --- |
|  |  | **2018** | **2025** | **2020** | **2025** | **2030** | **2020** | **2025** | **2030** |
| 1 | Orthopaedics &  Rheumatology | 158 | 253 | 213 | 349 | 513 | -1 | -96 | -241 |
| 2 | Respiratory Medicine | 48 | 166 | 253 | 334 | 386 | -108 | -167 | -237 |
| 3 | Paediatric Medicine | 355 | 445 | 429 | 534 | 682 | -5 | -89 | -227 |
| 4 | Obstetrics | 371 | 494 | 414 | 584 | 727 | 23 | -90 | -225 |
| 5 | Gastroenterology | 68 | 125 | 102 | 202 | 335 | -10 | -77 | -189 |
| 6 | Psychiatry | 160 | 259 | 338 | 367 | 403 | -78 | -108 | -163 |
| 7 | Cardiology &  Cardiothoracic | 285 | 389 | 318 | 444 | 539 | 40 | -55 | -153 |
| 8 | General Surgery | 314 | 415 | 361 | 458 | 554 | 27 | -43 | -142 |
| 9 | Oncology &  Haematology | 31 | 80 | 116 | 147 | 178 | -43 | -67 | -103 |
| 10 | Renal Medicine | 23 | 53 | 48 | 93 | 155 | -10 | -41 | -93 |
| 11 | Paediatric Surgery | 60 | 90 | 85 | 127 | 174 | -3 | -37 | -81 |
| 12 | Urology | 39 | 58 | 39 | 76 | 134 | 7 | -18 | -65 |
| 13 | Immunology &  Infections | 28 | 50 | 64 | 75 | 85 | -16 | -25 | -39 |
| 14 | Neonatology & NICU | 614 | 691 | 592 | 658 | 709 | 100 | 33 | -33 |
| 15 | Rheumatology | 13 | 25 | 6 | 9 | 13 | -7 | -15 | -29 |
| 16 | Transplantation | 0 | 4 | 2 | 3 | 10 | -3 | -5 | -9 |
| 17 | ENT; Head & Neck | 46 | 54 | 36 | 48 | 56 | 16 | 6 | -2 |
| 18 | vascular Surgery | 40 | 50 | 22 | 35 | 50 | 25 | 15 | 0 |
| 19 | Dentistry | 4 | 5 | 4 | 5 | 5 | 0 | 0 | 0 |
| 20 | Dermatology | 9 | 10 | 4 | 6 | 7 | 6 | 4 | 3 |
| 21 | Ophthalmology | 23 | 25 | 9 | 12 | 14 | 15 | 13 | 10 |
| 22 | Neurology | 88 | 106 | 51 | 74 | 88 | 51 | 32 | 16 |
| 23 | Plastic Surgery | 56 | 64 | 28 | 34 | 32 | 35 | 30 | 29 |
| 24 | Burns | 62 | 64 | 7 | 10 | 12 | 57 | 54 | 52 |
| 25 | Endocrinology | 112 | 125 | 53 | 62 | 68 | 72 | 64 | 55 |
| 26 | Neurosurgery | 104 | 113 | 32 | 38 | 43 | 81 | 75 | 69 |
| 27 | General Medicine | 485 | 546 | 329 | 398 | 452 | 208 | 148 | 85 |
| 28 | Gynaecology | 213 | 221 | 49 | 86 | 123 | 169 | 135 | 99 |

#### 6.2 Acute Inpatient care (Same Day)

The acute same day place supply of 1,086 places consists of all designated medical and surgical same day beds as well as treatment chairs for chemotherapy, infusions and renal dialysis. The distribution of this supply to specialties is based on acute overnight bed as reported by the survey and adjusted for local utilization figures calculated from HS17 activity data.

The gap analysis shows that Dubai has an undersupply of **41** acute same day places in 2020, which will increase to

**1,575** places by 2030.

Figure 16: Acute same day place gap, 2018 to 2030

3,000


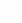


2,000

1,000

Supply Demand Gap Oversupply

###### 2,661

0


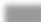

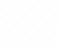


**1,800**

**1,086**

**722**

**1,086 1,127**

**1,086**

**1,086**

**364**

**-41**

**-714**

**Places**

-1,000

-2,000

**-1,575**

2018 2020 2025 2030

The gap analysis for each specialty shows that the top 5 specialties in requirement by 2030 will be Dialysis, Paediatric Medicine, Gastroenterology, Orthopaedics & Rheumatology and General Medicine.

Table 14: Acute same day place gap by specialty, 2018 to 2030

| **Specialty** | | **Supply** | **Demand** | | | **Gap** | | |
| --- | --- | --- | --- | --- | --- | --- | --- | --- |
|  |  | **2018** | **2020** | **2025** | **2030** | **2020** | **2025** | **2030** |
| 1 | Dialysis | 123 | 162 | 298 | 493 | -39 | -175 | -370 |
| 2 | Paediatric Medicine | 105 | 120 | 177 | 264 | -15 | -71 | -159 |
| 3 | Gastroenterology | 27 | 49 | 102 | 181 | -22 | -75 | -155 |
| 4 | Orthopaedics & Rheumatology | 43 | 65 | 118 | 188 | -21 | -75 | -144 |
| 5 | General Medicine | 167 | 191 | 248 | 296 | -24 | -81 | -129 |
| 6 | General Surgery | 92 | 117 | 152 | 190 | -25 | -60 | -98 |
| 7 | Haematology & Oncology | 9 | 53 | 75 | 103 | -44 | -65 | -94 |
| 8 | Cardiology & Cardiothoracic | 23 | 49 | 84 | 136 | 4 | -31 | -83 |
| 9 | Paediatric Surgery | 8 | 41 | 65 | 97 | -18 | -42 | -74 |
| 10 | Renal Medicine | 53 | 16 | 37 | 73 | -8 | -29 | -65 |
| 11 | Chemotherapy | 7 | 21 | 41 | 68 | 10 | -10 | -37 |
| 12 | Urology | 38 | 11 | 26 | 48 | 1 | -14 | -37 |
| 13 | Obstetrics | 12 | 27 | 50 | 73 | 11 | -12 | -35 |
| 14 | Gynaecology | 31 | 61 | 117 | 165 | 71 | 15 | -34 |
| 15 | Ophthalmology | 131 | 20 | 32 | 45 | -4 | -16 | -29 |
| 16 | Dentistry | 16 | 18 | 21 | 22 | -15 | -18 | -19 |
| 17 | Respiratory Medicine | 3 | 12 | 23 | 35 | 4 | -6 | -19 |
| 18 | Psychiatry | 16 | 17 | 21 | 25 | -6 | -9 | -14 |
| 19 | ENT; Head & Neck | 11 | 11 | 19 | 28 | 4 | -4 | -13 |
| 20 | Neurology | 16 | 14 | 26 | 40 | 17 | 5 | -9 |
| 21 | Immunology & Infections | 3 | 6 | 8 | 11 | -2 | -4 | -7 |
| 22 | vascular Surgery | 31 | 3 | 6 | 9 | 4 | 1 | -2 |
| 23 | Dermatology | 7 | 2 | 3 | 4 | 1 | 0 | -1 |
| 24 | Neonatology & NICU | 5 | 4 | 4 | 4 | 1 | 1 | 1 |
| 25 | Endocrinology | 9 | 16 | 22 | 30 | 19 | 12 | 5 |
| 26 | Burns | 34 | 1 | 1 | 2 | 8 | 8 | 7 |
| 27 | Neurosurgery | 25 | 7 | 10 | 13 | 18 | 16 | 12 |
| 28 | Plastic Surgery | 43 | 12 | 17 | 16 | 31 | 26 | 27 |

#### 6.3 Outpatient care (Consultation rooms)

There are a reported 5,160 outpatient consultation rooms in Dubai across the facility settings of hospitals, clinics and centres. The vast majority (70%) of these rooms are in Sector 3, with Sector 1 and 2 contributing 13% and 10% respectively.

The demand profile for outpatient care has been adjusted for local utilisation factors as derived from Health Statistics 2017 activity data, which gives a locally sensitive gap result based on current practices and models of care.

The gap analysis shows that Dubai is currently well served with outpatient consultation rooms with a requirement for an additional **447** rooms by 2025, increasing to **2,106** rooms by 2030.

Figure 17: Outpatient room gap, 2018 to 2030

Supply Demand Gap Oversupply


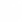


8,000


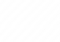

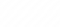


###### 5,160 5,160 5,160 5,607 7,266

6,000

4,000

**Rooms**

2,000

0

-2,000

###### 3,391 3,999

**1,769 1,161**

**-447**

**5,160**

-4,000

**-2,106**

2018 2020 2025 2030

The largest contributor to the gap in outpatient consultation rooms occurs in both the primary care and adult hospital outpatient service modes, with most clinic-based rooms being in oversupply up to 2030.

Figure 18: Outpatient room gap by service mode, 2018 to 2030

2018 2020 2025 2030 Oversupply


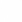


1,500

1,000


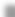

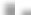

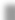

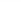

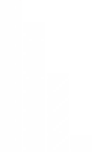

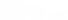

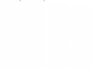

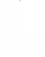


500

**Outpatient rooms**

0

**-101**

**-501-198**

**-726**

**-83**

**-120**

**-49**

**-93**

**-72**

**-213**

-500

**-474**

**-194**

**-262**

**- 218**

**-**

**322**

-1,000

**- 1,112**

-1,500

Adult Clinic

Paediatric Clinic

Primary Care Clinic

Womens Health Clinic

Adult Hospital

Allied Health Hospital

Paediatric Hospital

Womens Health Hospital

when viewing the gap by geographical sector, the large concentration of supply in Sector 3 results in no requirement of consultation rooms up to 2025, with an additional 105 rooms required by 2030. The majority of gap will occur in Sectors 1, 2, 4, 5 and 6, highlighting the current patient flow trends and historical focal capacity allocation in Sector

3. The geographic distribution of primary care may emerge as a significant concern with the increasing density of Dubai’s population and the associated increased travel times.

Figure 19: Outpatient room gap by sector, 2018 to 2030

2018 2020 2025 2030 Oversupply


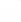


2000

1500


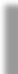

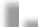

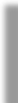

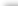

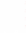

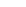

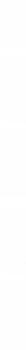

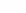

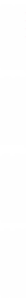

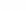

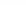

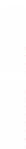


1000

**Outpatient rooms**

500

0

**- 77**

**- 105**

**- 11**

**- 22**

**- 54**

**- 89**

**- 211**

**- 51**

**- 139**

**- 7**

**- 9**

**- 15**

**- 21**

**- 15**

**- 22**

**- 43**

**- 65**

-500

**- 228**

**- 535**

**- 303**

**- 439**

**- 284**

**- 483**

**- 691**

-1000

1 2 3 4 5 6 7 8

Table 15: Outpatient room gap by specialty, 2018 to 2030, sorted by largest 2030 gap

| **Specialty** | | **Supply** | **Demand** | | | **Gap** | | |
| --- | --- | --- | --- | --- | --- | --- | --- | --- |
|  |  | **2018** | **2020** | **2025** | **2030** | **2020** | **2025** | **2030** |
| 1 | Primary Care | 677 | 875 | 1,178 | 1,403 | -198 | -501 | -726 |
| 2 | Allied Health | 101 | 221 | 295 | 363 | -120 | -194 | -262 |
| 3 | Paediatrics | 423 | 363 | 499 | 651 | 60 | -76 | -228 |
| 4 | General Medicine | 657 | 381 | 596 | 870 | 276 | 61 | -213 |
| 5 | Respiratory Medicine | 36 | 136 | 181 | 245 | -100 | -145 | -209 |
| 6 | Endocrinology | 97 | 143 | 207 | 286 | -46 | -110 | -189 |
| 7 | Neurology | 75 | 150 | 191 | 232 | -75 | -116 | -157 |
| 8 | Gastroenterology | 105 | 113 | 172 | 256 | -8 | -67 | -151 |
| 9 | Oncology & Haematology | 37 | 72 | 100 | 136 | -35 | -63 | -99 |
| 10 | Orthopaedics & Rheumatology | 349 | 236 | 315 | 419 | 113 | 34 | -70 |
| 11 | Immunology & Infections | 20 | 40 | 52 | 65 | -20 | -32 | -45 |
| 12 | Urology | 118 | 79 | 113 | 158 | 39 | 5 | -40 |
| 13 | Trauma and Injury | 8 | 28 | 36 | 42 | -20 | -28 | -34 |
| 14 | Renal Medicine | 26 | 29 | 38 | 53 | -3 | -12 | -27 |
| 15 | Dentistry | 55 | 40 | 57 | 80 | 15 | -2 | -25 |
| 16 | vascular Surgery | 17 | 19 | 26 | 34 | -2 | -9 | -17 |
| 17 | Neonatology | 2 | 6 | 8 | 10 | -4 | -6 | -8 |
| 18 | Psychiatry | 140 | 83 | 111 | 138 | 57 | 29 | 2 |
| 19 | Neurosurgery | 42 | 12 | 16 | 20 | 30 | 26 | 22 |
| 20 | ENT; Head & Neck | 183 | 90 | 116 | 149 | 93 | 67 | 34 |
| 21 | Obstetrics | 307 | 143 | 179 | 262 | 164 | 128 | 45 |
| 22 | General Surgery | 181 | 66 | 91 | 122 | 115 | 90 | 59 |
| 23 | Cardiology & Cardiothoracic | 193 | 61 | 91 | 131 | 132 | 102 | 62 |
| 24 | Ophthalmology | 218 | 62 | 90 | 140 | 156 | 128 | 78 |
| 25 | Plastic Surgery | 205 | 24 | 31 | 37 | 181 | 174 | 168 |
| 26 | Gynaecology | 515 | 114 | 170 | 247 | 401 | 345 | 268 |
| 27 | Dermatology | 373 | 69 | 81 | 93 | 304 | 292 | 280 |

#### 6.4 Emergency Department

Across Dubai, there are 501 Emergency bays of which 229 are allocated to Life Threatening care and 272 are allocated to Urgent Care. Most of these bays are in Sector 3 (61%), followed by Sector 1 (22%) and Sector 2 (10%).

The gap analysis indicates that Dubai is well served by total emergency bays currently and will require **35** bays by 2025, increasing to **107** bays by 2030. when the gap is analysed by type of bay, it shows an immediate requirement for Life Threatening bays, increasing from **26** bays currently to **211** bays by 2030. This presents an opportunity to repurpose existing capacity to meet this current and emerging requirement.

Figure 20: Supply, demand and gap for ED bays, 2018 to 2030

800

600

400

**ED Bays**

200

0

-200

Supply Demand Gap Oversupply

2018 2020 2025 2030


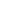

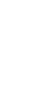

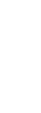

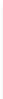

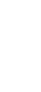

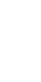

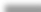

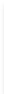

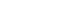

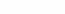


**501**

**401**

**511**

**451**

**521 556**

**521 628**

**100**

**60**

**-35**

**-107**

Figure 21: Gap by type, 2018 to 2030

2018 2020 2025 2030 Oversupply


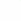


300

150


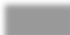

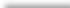

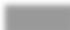

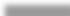

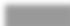

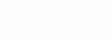

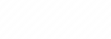

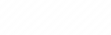

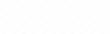


**-26**

**-59**

**-142**

**-211**

**ED Bays**

0

-150

-300

**126**

**118**

**107**

**105**

Life Theatening Urgent

#### 6.5 Procedural Care / Medical Imaging

The distribution of procedural care, or medical imaging, units amongst the Emirate is heavily focused in Sector 3 (65%), Sector 1 (14%) and Sector 2 (12%). The demand profile estimates for each procedural care unit type has local relative utilisation rates applied where indicated after Expert Panel discussion; this was most pronounced for SPECT/SPECT-CT, PET and Radiation therapy.

The gap analysis for procedural care units is summarised in the table below, followed by accompanying supply, demand and gap figures for each unit type.

Table 16: Procedural care unit gap by type, 2018 to 2030

| **Specialty** | | **Supply** | **Demand** | | | **Gap** | | |
| --- | --- | --- | --- | --- | --- | --- | --- | --- |
|  |  | **2018** | **2020** | **2025** | **2030** | **2020** | **2025** | **2030** |
| 1 | Ultrasound | 376 | 422 | 642 | 860 | -46 | -266 | -484 |
| 2 | Computed Tomography | 58 | 64 | 87 | 108 | -6 | -29 | -50 |
| 3 | Mammography | 57 | 70 | 88 | 100 | -13 | -31 | -43 |
| 4 | Angiography | 21 | 31 | 42 | 52 | -10 | -21 | -31 |
| 5 | SPECT/SPECT-CT | 7 | 16 | 21 | 25 | -9 | -14 | -18 |
| 6 | Magnetic Resonance | 59 | 39 | 56 | 72 | 20 | 3 | -13 |
| 7 | Positron Emission  Tomography | 5 | 2 | 4 | 5 | 2 | 1 | -1 |
| 8 | Radiation Therapy | 4 | 2 | 3 | 3 | 2 | 1 | 1 |
| 9 | x Ray | 334 | 162 | 222 | 273 | 172 | 112 | 62 |

1,000

**Ultrasound**

500

0

-500

-1,000

Supply Demand Gap Oversupply

**376**

**360**

**16**

**376**

**422**

**376**

**642**

**860**

**376**

**- 46**

**- 266**

**- 484**

2018 2020 2025 2030


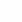


Supply Demand Gap Oversupply


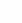


400


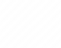

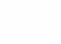

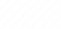

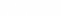


**334**

**334**

**334**

**334**

**144 190**

**162 172**

**222**

**273**

**112**

**61**

**X-ray**

200

0

2018 2020 2025 2030

Supply Demand Gap Oversupply


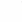


100 50

**21**

**27**

**21**

**31**

**21**

**42**

**52**

**- 6**

**- 10**

**- 21**

**- 31**

**21**

**Angiography**

0

-50

2018 2020 2025 2030

Supply Demand Gap Oversupply


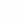


200

| **58 56 58 64 58** | | | | | | | |  | **58** | |  |  | | |
| --- | --- | --- | --- | --- | --- | --- | --- | --- | --- | --- | --- | --- | --- | --- |
|  |  |  | **2** |  |  |  |  |  |  |  |  |  |  |  |
|  | | | | | | | | | | | | |  |  |

100

**CT**

0

-100

**87 108**

**- 6 - 29 - 50**

2018 2020 2025 2030

Supply Demand Gap Oversupply


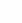


40

**7**

**15**

**7**

**16**

**21**

**25**

**- 8**

**- 9**

**- 14**

**- 18**

**7**

**7**

**SPECT/SPECT**

20

0

-20

2018 2020 2025 2030

100

50

**MRI**

0

Supply Demand Gap Oversupply

**72**


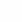


|  | **59** | **34 25** | | | **59** | **39 20** | | | **59** | **56** |  | **59** |  |  |
| --- | --- | --- | --- | --- | --- | --- | --- | --- | --- | --- | --- | --- | --- | --- |
|  |  |  | | |  |  |  | |  |  | **3** |  |  |  |
|  |  |  |  |  |  |  |  |  |  |  |  |  |  |  |


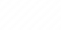

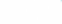


-50

**- 13**

2018 2020 2025 2030

Supply Demand Gap Oversupply


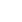


200

**Mammography**

| **57 64 57** | | | | | **70** | **57** | |  | **57** | | | |  |  | | |
| --- | --- | --- | --- | --- | --- | --- | --- | --- | --- | --- | --- | --- | --- | --- | --- | --- |
|  |  |  |  |  |  |  |  |  |  | | |  |  |  |  |  |
|  | | | | | | | | | |  |  | | | |  |  |

100

0

-100

**88 100**

**- 7 - 13 - 31 - 43**

2018 2020 2025 2030

Supply Demand Gap Oversupply


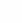


10

**5**

**5**

**5**

**4**

**5**

**5**

**1**

**3**

**2**

**3**

**2**

5

**PET**

0

-5 **- 1**

2018 2020 2025 2030

Supply Demand Gap Oversupply

**Radiation Therapy**

6

**4**

**4**

**4**

**4**

**2**

**2**

**2**

**2**

**3**

**3**

**1**

**1**

4

2

0

2018 2020 2025 2030

#### 6.6 Critical Care beds

There are currently 902 critical care beds in Dubai (526 Adult, 299 Neonatal and 77 Paediatric), with an additional planned supply of 144 adult ICU and 61 NICU beds expected to be commissioned by 2025.

The gap analysis indicates that critical care beds will remain oversupplied up to 2030, however when displayed by type of bed, there is an undersupply of **116** Neonatal ICU beds by 2025 which increases to **233** by 2030.

Supply Demand Gap Oversupply

1,000

**599**

**484**

**647**

**521**

**670**

**510**

**670**

**479**

**191**

**160**

**126**

**115**

**Adult ICU**

500

0

2018 2020 2025 2030

100

**Paediatric ICU**

Supply Demand Gap Oversupply

**77 77 77 77**

50 **28 49**

0

**32 45**

**47 54**

**23**

**30**

2018 2020 2025 2030

1,000

**Neonatal ICU**

500

0

-500

Supply Demand Gap Oversupply

**- 116 - 233**

**299**

**231**

**345**

**68**

**269**

**360**

**476**

**593**

**360**

**76**

2018 2020 2025 2030

#### 6.7 Non-Acute Care and Long-Term Care beds

The current supply of non-acute care beds is made up of 64 long term care beds and 71 rehabilitation beds, with a further 30 rehabilitation beds planned by 2020. The demand profile for non-acute care has been adjusted for local utilisation factors, with only 10% of non-national population demand being considered needed.

The gap analysis indicates that non-acute care beds are currently in undersupply and their future requirements will continue to grow, increasing from **214** beds in 2018 to **675** beds by 2030.

Figure 22: Supply, demand and gap for Non-acute care beds, 2018 to 2030

1,000

**Non-acute beds**

500

0

-500

-1,000

**135**

**349**

Supply Demand Gap Oversupply

**397 598 820**

**145 145 145**

**- 214 - 252 - 453**

**- 675**

2018 2020 2025 2030

when analysed by type of bed, the largest contributor to non-acute care bed gap is Rehabilitation, which currently requires 168 beds and will increase to 483 beds by 2030.

Figure 23: Figure 23 Gap by bed type, 2018 to 2030

2018 2020 2025 2030

0

**Non-acute beds**

-250

**- 46**

**- 60**

**- 122**

**- 192**

**- 168**

**- 192**

-500

**- 331**

**- 483**

-750

LTC Rehabilitation

#### 6.8 Operating Theatres

There are currently 224 operating theatres within Dubai, of which 189 are used for elective surgeries and 35 for emergency surgeries. Based on planned facility licensing, an additional 23 operating theatres are expected by 2025.

Gap analysis of operating theatres indicates that there is an oversupply of operating theatres in Dubai which becomes an undersupply of 15 theatres in 2030.

Figure 24: Supply, demand and gap for Operating Theatres, 2018 to 2030

300

**Operating Theatres**

200

100

0

-100

Supply Demand Gap Oversupply

**224**

**236**

**247**

**247**

**262**

**154**

**169**

**221**

**70**

**67**

**26**

**- 15**

2018 2020 2025 2030

This is as a result of the oversupply of elective theatres, which does not reach a requirement for additional capacity in the next 12 years. whereas, additional emergency theatres are required by 2020 and is expected to increase to 25 theatres by 2030.

Figure 25: Gap by theatre type, 2018 to 2030

2018 2020 2025 2030 Oversupply

100

**84**

**68**

**38**

**11**

**3**

**- 1**

**- 12**

**- 25**

**Operating Theatres**

50

0

-50

Elective Emergency

Figure 26: Medical workforce gap by FTE, 2018 to 2030

Supply Demand Gap Oversupply

20,000

**16,271**

**11,958**

**7,842 7,616**

**7,842**

**8,599**

**7,842**

**226**

**- 757**

**- 4,116**

**7,842**

10,000

**Medical FTE**

0

-10,000

**- 8,429**

2018 2020 2025 2030

Medical workforce requirements by specialty are shown below and have been derived from demand growth in inpatient and outpatient activity for each specialty.

Figure 27: Medical specialty workforce gap analysis, 2020 to 2030

| **2020** | | | **2030** | | |
| --- | --- | --- | --- | --- | --- |
| **Medical specialty** | **Supply** | **Gap** | **Medical specialty** | **Supply** | **Gap** |
| **General Medicine and Surgery** | 1,624 | -1,034 | **General Medicine and Surgery** | 1,624 | -4,184 |
| **Paediatrics** | 635 | -726 | **Paediatrics** | 635 | -2,144 |
| **Anaesthetics** | 353 | -166 | **General Medical Practice** | 1,687 | -472 |
| **Endocrinology** | 70 | -62 | **Anaesthetics** | 353 | -455 |
| **Psychiatry** | 75 | -59 | **Obstetrics** | 574 | -227 |
| **Haematology** | 11 | -58 | **Endocrinology** | 70 | -213 |
| **Rehabilitation** | 25 | -45 | **Cardiology** | 130 | -189 |
| **Immunology & Infections** | 17 | -40 | **Renal Medicine** | 43 | -142 |
| **Dentistry** | 25 | -32 | **Respiratory Medicine** | 51 | -127 |
| **Respiratory Medicine** | 51 | -30 | **Critical Care Medicine** | 78 | -127 |
| **Cardiology** | 130 | -21 | **Neurology** | 87 | -103 |
| **Renal Medicine** | 43 | -20 | **Orthopaedics** | 388 | -102 |
| **Neurology** | 87 | -19 | **Psychiatry** | 75 | -90 |
| **Critical Care Medicine** | 78 | -17 | **Immunology & Infections** | 17 | -87 |
| **Clinical Genetics** | 3 | -14 | **Haematology** | 11 | -87 |
| **Audiological Medicine** | 3 | -11 | **Rehabilitation** | 25 | -85 |
| **Nuclear Medicine** | 8 | -10 | **Dentistry** | 25 | -77 |
| **Oncology** | 24 | -6 | **Ophthalmology** | 217 | -62 |
| **Cardiothoracic Surgery** | 32 | -1 | **Oncology** | 24 | -61 |
|  |  |  | **Radiology** | 258 | -57 |
|  |  |  | **Audiological Medicine** | 3 | -29 |
|  |  |  | **Neonatology** | 43 | -28 |
|  |  |  | **Cardiothoracic Surgery** | 32 | -27 |
|  |  |  | **Clinical Genetics** | 3 | -26 |
|  |  |  | **Interventional Cardiology** | 52 | -23 |
|  |  |  | **Nuclear Medicine** | 8 | -16 |
|  |  |  | **Radiation Oncology** | 17 | -15 |
|  |  |  | **Rheumatology** | 25 | -5 |

There are currently 16,335 FTE nurses licensed in Dubai, with a current shortfall of 388 FTEs, increasing to nearly 14,000 by 2030. The growth in nurse demand is in keeping with the activity growth in overnight, same day and ambulatory health care services within the Emirate.

Figure 28: Nursing workforce gap by FTE

Supply Demand Gap

45,000

**25,404**

**30,258**

**16,335 16,723**

**16,335 18,826**

**16,335**

**- 388**

**- 2,491**

**- 9,069**

**- 13,923**

**16,335**

30,000

**Nursing FTE**

15,000

0

-15,000

-30,000

2018 2020 2025 2030

Table 17: Summary of gaps by KPU, 2018 to 2030

| **Key Planning Unit** | **Supply** | **Demand** | | | **Gap** | | |
| --- | --- | --- | --- | --- | --- | --- | --- |
|  | **2018** | **2020** | **2025** | **2030** | **2020** | **2025** | **2030** |
| **Acute overnight beds** | 3,795 | 4,002 | 5,268 | 6,547 | 654 | -311 | -1,590 |
| **Acute same day beds** | 1,086 | 1,127 | 1,800 | 2,661 | -41 | -714 | -1575 |
| **Outpatient consultation rooms** | 5,160 | 3,999 | 5,607 | 7,266 | 1,161 | -447 | -2,106 |
| **Non-Acute overnight beds** | 135 | 397 | 598 | 820 | -252 | -453 | -675 |
| **Emergency department bays** | 501 | 451 | 556 | 628 | 60 | -35 | -107 |
| **CC – Adult ICU** | 599 | 126 | 160 | 191 | 521 | 510 | 479 |
| **CC – Paediatric ICU** | 77 | 32 | 47 | 54 | 45 | 30 | 23 |
| **CC – Neonatal ICU** | 299 | 269 | 476 | 593 | 76 | -116 | -233 |
| **Operating Theatres (Elective)** | 189 | 133 | 174 | 201 | 68 | 38 | 11 |
| **Operating Theatres (Emergency)** | 35 | 36 | 47 | 60 | -1 | -12 | -25 |
| **Human Resources - Medical** | 7,842 | 8,599 | 11,958 | 16,271 | -465 | -4,053 | -7,067 |
| **Human Resources - Nursing** | 16,335 | 18,826 | 25,404 | 30,258 | -2,491 | -9,069 | -13,923 |
| **Ultrasound** | 376 | 372 | 572 | 765 | -46 | -266 | -484 |
| **Computed Tomography** | 58 | 56 | 77 | 95 | -6 | -29 | -50 |
| **Mammography** | 57 | 59 | 76 | 86 | -13 | -31 | -43 |
| **Angiography** | 21 | 29 | 41 | 50 | -10 | -21 | -31 |
| **SPECT/SPECT-CT** | 7 | 15 | 20 | 23 | -9 | -14 | -18 |
| **Magnetic Resonance** | 59 | 36 | 52 | 67 | 20 | 3 | -13 |
| **Positron Emission Tomography** | 5 | 2 | 3 | 5 | 2 | 1 | -1 |
| **Radiation Therapy** | 4 | 2 | 2 | 3 | 2 | 1 | 1 |
| **X Ray** | 334 | 138 | 191 | 234 | 172 | 112 | 62 |

Note: Gap numbers highlighted in red colour with a –ve sign represents an undersupply

Table 18: “Traffic light” assessment for supply gaps

Gap/Supply Priority Description

**>50%**

**25-50%**

**0-25%**

**<0%**

**High** > 50% deficit in total supply gap **Medium** 25-50% deficit in total supply gap **Low** 0-25% deficit in total supply gap

**No** No deficit in total supply gap

The priority table highlights both the gap as a proportion of current supply and the actual gap for each specialty by key planning unit for 2020 and 2030, indicating the priority of planning and investment actions for each service specialty.

Table 19: Acute overnight priorities by specialty, shown by gap percentage and number, 2020-30

| **Acute overnight beds** | | | | | |
| --- | --- | --- | --- | --- | --- |
| **2020** | | | **2030** | | |
| % | No. | Specialty | % | No. | Specialty |
| -74% | -108 | Respiratory Medicine | >100% | -237 | Respiratory Medicine |
| -59% | -43 | Haematology & Oncology | >100% | -93 | Renal Medicine |
| -33% | -16 | Immunology & Infections | >100% | -103 | Haematology & Oncology |
| -30% | -78 | Psychiatry | >100% | -189 | Gastroenterology |
| -27% | -10 | Renal Medicine | -95% | -65 | Urology |
| -11% | -10 | Gastroenterology | -88% | -240 | Orthopaedics & Rheumatology |
| -3% | -3 | Paediatric Surgery | -86% | -81 | Paediatric Surgery |
| -1% | -5 | Paediatric Medicine | -82% | -39 | Immunology & Infections |
| -1% | -1 | Orthopaedics & Rheumatology | -68% | -163 | Psychiatry |
| 5% | 23 | Obstetrics | -50% | -227 | Paediatric Medicine |
| 7% | 27 | General Surgery | -45% | -225 | Obstetrics |
| 11% | 40 | Cardiology & Cardiothoracic | -40% | -153 | Cardiology & Cardiothoracic |
| 14% | 100 | Neonatology & NICU | -34% | -142 | General Surgery |
| 15% | 7 | Urology | -5% | -33 | Neonatology & NICU |
| 18% | 1 | Dentistry | -4% | -2 | ENT; Head & Neck |
| 31% | 16 | ENT; Head & Neck | 0% | 0 | vascular Surgery |
| 39% | 208 | General Medicine | 0% | 0 | Dentistry |
| 50% | 51 | Neurology | 15% | 16 | Neurology |
| 53% | 25 | vascular Surgery | 16% | 85 | General Medicine |
| 55% | 35 | Plastic Surgery | 27% | 3 | Dermatology |
| 57% | 72 | Endocrinology | 41% | 10 | Ophthalmology |
| 59% | 6 | Dermatology | 45% | 55 | Endocrinology |
| 63% | 15 | Ophthalmology | 45% | 99 | Gynaecology |
| 71% | 81 | Neurosurgery | 47% | 29 | Plastic Surgery |
| 78% | 169 | Gynaecology | 62% | 69 | Neurosurgery |
| 90% | 57 | Burns | 82% | 52 | Burns |

Table 20: Acute same day priorities by specialty, shown by gap percentage and number, 2020-30

| **Acute same day beds** | | | | | |
| --- | --- | --- | --- | --- | --- |
| **2020** | | | **2030** | | |
| % | No. | Specialty | % | No. | Specialty |
| >100% | -44 | Haematology & Oncology | >100% | -370 | Dialysis |
| >100% | -15 | Dentistry | >100% | -159 | Paediatric Medicine |
| -98% | -8 | Renal Medicine | >100% | -155 | Gastroenterology |
| -84% | -22 | Gastroenterology | >100% | -144 | Orthopaedics & Rheumatology |
| -78% | -18 | Paediatric Surgery | >100% | -98 | General Surgery |
| -59% | -2 | Immunology & Infections | >100% | -94 | Haematology & Oncology |
| -53% | -6 | Psychiatry | >100% | -83 | Cardiology & Cardiothoracic |
| -49% | -21 | Orthopaedics & Rheumatology | >100% | -74 | Paediatric Surgery |
| -32% | -39 | Dialysis | >100% | -65 | Renal Medicine |
| -27% | -25 | General Surgery | >100% | -37 | Urology |
| -25% | -4 | Ophthalmology | >100% | -37 | Chemotherapy |
| -15% | -24 | General Medicine | >100% | -29 | Ophthalmology |
| -14% | -15 | Paediatric Medicine | >100% | -19 | Dentistry |
| 6% | 1 | Urology | >100% | -19 | Respiratory Medicine |
| 7% | 4 | Cardiology & Cardiothoracic | >100% | -14 | Psychiatry |
| 19% | 1 | Dermatology | >100% | -7 | Immunology & Infections |
| 21% | 1 | Neonatology & NICU | -94% | -35 | Obstetrics |
| 25% | 4 | Respiratory Medicine | -81% | -13 | ENT; Head & Neck |
| 29% | 4 | ENT; Head & Neck | -77% | -129 | General Medicine |
| 29% | 11 | Obstetrics | -31% | -1 | Dermatology |
| 31% | 10 | Chemotherapy | -30% | -2 | vascular Surgery |
| 54% | 71 | Gynaecology | -29% | -9 | Neurology |
| 54% | 19 | Endocrinology | -26% | -34 | Gynaecology |
| 55% | 17 | Neurology | 14% | 5 | Endocrinology |
| 57% | 4 | vascular Surgery | 19% | 1 | Neonatology & NICU |
| 71% | 31 | Plastic Surgery | 49% | 12 | Neurosurgery |
| 73% | 18 | Neurosurgery | 63% | 27 | Plastic Surgery |
| 93% | 8 | Burns | 74% | 7 | Burns |

Table 21: Outpatient priorities by specialty, shown by gap percentage and number, 2020-30

| **Outpatient consultation rooms** | | | | | |
| --- | --- | --- | --- | --- | --- |
| **2020** | | | **2030** | | |
| % | No. | Specialty | % | No. | Specialty |
| >100% | -100 | Respiratory Medicine | >100% | -209 | Respiratory Medicine |
| >100% | -20 | Trauma and Injury | >100% | -34 | Trauma and Injury |
| >100% | -4 | Neonatology | >100% | -8 | Neonatology |
| >100% | -120 | Allied Health | >100% | -99 | Oncology & Haematology |
| >100% | -75 | Neurology | >100% | -262 | Allied Health |
| -98% | -20 | Immunology & Infections | >100% | -45 | Immunology & Infections |
| -93% | -35 | Oncology & Haematology | >100% | -157 | Neurology |
| -47% | -46 | Endocrinology | >100% | -189 | Endocrinology |
| -29% | -198 | Primary Care | >100% | -151 | Gastroenterology |
| -13% | -2 | vascular Surgery | >100% | -726 | Primary Care |
| -12% | -3 | Renal Medicine | >100% | -27 | Renal Medicine |
| -8% | -8 | Gastroenterology | -97% | -17 | vascular Surgery |
| 14% | 60 | Paediatrics | -54% | -228 | Paediatrics |
| 27% | 15 | Dentistry | -46% | -25 | Dentistry |
| 33% | 113 | Orthopaedics & Rheumatology | -34% | -40 | Urology |
| 33% | 39 | Urology | -32% | -213 | General Medicine |
| 41% | 57 | Psychiatry | -20% | -70 | Orthopaedics & Rheumatology |
| 42% | 276 | General Medicine | 1% | 2 | Psychiatry |
| 51% | 93 | ENT; Head & Neck | 15% | 45 | Obstetrics |
| 53% | 164 | Obstetrics | 18% | 34 | ENT; Head & Neck |
| 63% | 115 | General Surgery | 32% | 62 | Cardiology & Cardiothoracic |
| 69% | 132 | Cardiology & Cardiothoracic | 33% | 59 | General Surgery |
| 71% | 30 | Neurosurgery | 36% | 78 | Ophthalmology |
| 72% | 156 | Ophthalmology | 51% | 22 | Neurosurgery |
| 78% | 401 | Gynaecology | 52% | 268 | Gynaecology |
| 81% | 304 | Dermatology | 75% | 280 | Dermatology |
| 88% | 181 | Plastic Surgery | 82% | 168 | Plastic Surgery |

**TAHPI**
